# Therapeutic Potential of *EWSR1–FLI1* Inactivation by CRISPR/Cas9 in Ewing Sarcoma

**DOI:** 10.3390/cancers13153783

**Published:** 2021-07-27

**Authors:** Saint T. Cervera, Carlos Rodríguez-Martín, Enrique Fernández-Tabanera, Raquel M. Melero-Fernández de Mera, Matias Morin, Sergio Fernández-Peñalver, Maria Iranzo-Martínez, Jorge Amhih-Cardenas, Laura García-García, Laura González-González, Miguel Angel Moreno-Pelayo, Javier Alonso

**Affiliations:** 1 Unidad de Tumores Sólidos Infantiles, Instituto de Investigación de Enfermedades Raras (IIER), Instituto de Salud Carlos III (ISCIII), 28220 Madrid, Spain; scervera@isciii.es (S.T.C.); carlosrm@isciii.es (C.R.-M.); efernandezt@externos.isciii.es (E.F.-T.); raquel.melero@externos.isciii.es (R.M.M.-F.d.M.); mariairanzom@gmail.com (M.I.-M.); jorge.amhih@isciii.es (J.A.-C.); garciagarcialaura83@gmail.com (L.G.-G.); lauragg911@gmail.com (L.G.-G.); 2 Centro de Investigación Biomédica en Red de Enfermedades Raras, Instituto de Salud Carlos III (CB06/07/1009; CIBERER-ISCIII), 28029 Madrid, Spain; 3 Servicio de Genética, Hospital Universitario Ramón y Cajal, IRYCIS, Carretera de Colmenar km 9.100, 28034 Madrid, Spain; matias.morin@salud.madrid.org (M.M.); sergio.fernandez@hrc.es (S.F.-P.); mmorenop@salud.madrid.org (M.A.M.-P.); 4 Centro de Investigación Biomédica en Red de Enfermedades Raras, Instituto de Salud Carlos III (CB06/07/0048; CIBERER-ISCIII), 28029 Madrid, Spain

**Keywords:** Ewing sarcoma, EWSR1–FLI1, CRISPR/Cas9, gene therapy, cell cycle arrest, senescence

## Abstract

**Simple Summary:**

Ewing sarcoma is an aggressive tumor with still unacceptable survival rates, particularly in patients with metastatic disease and for which it is necessary to develop new and innovative therapies. These tumors are characterized by the presence of chromosomal translocations that give rise to chimeric transcription factors (i.e., EWSR1–FLI1) that govern the oncogenic process. In this article, we describe an efficient strategy to permanently inactivate the EWSR1–FLI1 oncogene characteristic of Ewing sarcoma using CRISPR/Cas9 gene editing technology. Although the application of gene therapy in cancer still has many limitations, for example, the strategy for delivery, studies like ours show that gene therapy can be a promising alternative, particularly for those tumors that are highly dependent on a particular oncogene as is the case in Ewing sarcoma.

**Abstract:**

Ewing sarcoma is an aggressive bone cancer affecting children and young adults. The main molecular hallmark of Ewing sarcoma are chromosomal translocations that produce chimeric oncogenic transcription factors, the most frequent of which is the aberrant transcription factor EWSR1–FLI1. Because this is the principal oncogenic driver of Ewing sarcoma, its inactivation should be the best therapeutic strategy to block tumor growth. In this study, we genetically inactivated EWSR1–FLI1 using CRISPR-Cas9 technology in order to cause permanent gene inactivation. We found that gene editing at the exon 9 of FLI1 was able to block cell proliferation drastically and induce senescence massively in the well-studied Ewing sarcoma cell line A673. In comparison with an extensively used cellular model of EWSR1–FLI1 knockdown (A673/TR/shEF), genetic inactivation was more effective, particularly in its capability to block cell proliferation. In summary, genetic inactivation of EWSR1–FLI1 in A673 Ewing sarcoma cells blocks cell proliferation and induces a senescence phenotype that could be exploited therapeutically. Although efficient and specific in vivo CRISPR-Cas9 editing still presents many challenges today, our data suggest that complete inactivation of EWSR1–FLI1 at the cell level should be considered a therapeutic approach to develop in the future.

## 1. Introduction

Ewing sarcoma is a highly aggressive rare bone and soft tissue tumor arising in children and young adults [1]. Despite international collaborative efforts to improve Ewing sarcoma outcome [2,3,4], overall survival rates for patients with localized disease have remained stagnant at approximately 65–70% for decades, and the situation is especially frustrating for relapsed patients whose prognosis is certainly fatal [5]. Hence, it is necessary to identify innovative therapies that represent a real breakthrough in treating this disease.

The biology of Ewing sarcoma is relatively well known. From a molecular perspective, Ewing sarcoma is characterized by reciprocal chromosomal translocations that give rise to fusion proteins that govern tumorigenesis [1]. The most frequent of these fusions proteins is EWSR1–FLI1 in which the N-terminal region of the EWSR1 gene is fused to the C-terminal region of the transcription factor FLI1 containing the DNA binding domain [6]. EWSR1–FLI1 results from the chromosomal translocation t(11;22), present in nearly 85% of Ewing sarcoma tumors. The remaining tumors harbor other functionally equivalent chromosomal translocations involving other transcription factors (i.e., ERG, FEV) and other EWSR1-related proteins (i.e., FUS) [1]. In contrast, other chromosomal aberrations are rare and point mutations affecting oncogenes or tumor suppressor genes are even less frequent [7,8]. In light of this data, it is thoroughly accepted that these chimeric transcription factors are the main oncogenic drivers in Ewing sarcoma [1].

Experimental data accumulated over the last 20 years have demonstrated that EWSR1–FLI1 plays a central role in Ewing sarcoma pathogenesis. As an aberrant transcription factor, EWSR1–FLI1 dictates a specific transcriptional program that promotes cell proliferation and blocks cell differentiation [9,10]. Several direct and indirect EWSR1–FLI1 targets contributing to this transcriptional program have been identified, and functional studies have corroborated its individual contribution to cancer pathogenesis [11,12,13,14,15]. In addition, EWSR1–FLI1 also contributes to defining an epigenetic program in Ewing sarcoma cells that also contributes to the oncogenic profile [16,17,18]. Nevertheless, the most relevant data demonstrating the critical role of EWSR1–FLI1 in Ewing sarcoma pathogenesis comes from functional studies carried out in Ewing sarcoma cell models in which EWSR1–FLI1 expression has been knocked-down using RNA interference approaches. Those studies have shown that EWSR1–FLI1 knockdown impairs proliferation of Ewing sarcoma cells both in vitro and in vivo, reinforcing the idea that EWSR1–FLI1 targeted inactivation should be the most effective therapy against Ewing sarcoma [19].

The discovery of the CRISPR/Cas9 system in bacteria and its rapid incorporation into laboratories as a tool for biochemical and genetic manipulation has definitively changed the way research is conducted and has opened a wide range of therapeutic opportunities [20,21,22]. Cas9 is a nuclease that produces double-stranded breaks (DSBs) at specific DNA localizations guided by an RNA sequence targeting a particular region [23]. The guide RNA has minimal requirements, the most important of which is a sequence located in the DNA, immediately juxtaposed to the sequence recognized by the RNA guide, called protospacer adjacent motif (PAM). Since PAM sequences range from 2 to 8 nucleotides depending on each nuclease, several possible guide RNAs can be located in a determined gene [24]. The efficiency of Cas9-mediated DSBs depends on the specific requirements of the guide RNA sequence, some of which have been identified [25]. However, determining the specificity and efficiency of a particular guide RNA can ultimately only be assessed experimentally. 

If a donor DNA accompanies Cas9 and guide RNA, the DNA target sequence will be modified according to this donor DNA thanks to the homology-directed repair (HDR) system present in the cells. The HDR system will repair the double-stranded breaks produced by the Cas9 nuclease using the donor DNA as a template. However, the primary autonomous cell system involved in repairing the DSBs generated by Cas9 is the non-homologous end joining (NHEJ). According to this mechanism, DNA ends are directly ligated without the need for a homologous template. This DNA repairing pathway is error-prone, generating short deletions or insertions and a defective repair of DNA double-strand breaks. If this happens in coding DNA, it will produce frameshift mutations and, consequently, premature stop codons and non-functional proteins. The easiness of producing null alleles using CRISPR libraries has been utilized to identify essential genes in cancer cell lines targeting hundreds of thousands of genes [26,27,28], to produce more easily loss of function animal models [29], or to inactivate oncogenes in human cancer cells both in vitro and in vivo [30,31].

In this study, we used CRISPR/Cas9 technology to inactivate genetically the more frequent fusion gene in Ewing sarcoma, EWSR1–FLI1, and analyzed the cellular and molecular implications of gene inactivation in comparison to gene silencing. We identified, experimentally, a guide RNA that specifically and efficiently targets the EWSR1–FLI1 gene and demonstrate that this strategy produces full cell cycle arrest and senescence that could be exploited therapeutically. 

## 2. Materials and Methods

### 2.1. Cell Lines

The Ewing sarcoma cell lines A673 (CRL-1598) were purchased from American Type Culture Collection (Manassas, VA, USA) and maintained in Dulbecco’s modified Eagle’s medium (DMEM) supplemented with 10% fetal bovine serum (FBS), 50 U/mL penicillin, and 50 µg/mL streptomycin. The A673/TR/shEF cells, which express a specific shRNA inducible by doxycycline directed against EWSR1–FLI1 mRNA, have previously been described in detail [12]. The A673/TR/shEF cells were maintained in DMEM supplemented with 10% tetracycline-free FBS (Capricorn Scientific, Ebsdorfergrund, Germany), 50 U/mL penicillin, 50 µg/mL streptomycin, 100 μg/mL zeocin, and 3 μg/mL blasticidin. The A673/TR/shEF cells were stimulated with doxycycline (1 µg/mL) to induce the expression of EWSR1–FLI1-specific shRNA.

### 2.2. Establishment of A673 Cells Expressing Cas9 Protein and sgRNAs

The A673 cells were infected with lentiviral particles carrying CP-LvC9NU-02 vector (Genecopoeia, Rockville, MD, USA) encoding Cas9 and GFP under the CMV and SV40 promoters, respectively. Cells were infected using two different (2.5 and 5) multiplicity of infection (MOI) and selected with G418 (500 µg/mL). Clonal cell populations were obtained by the dilution limit method. Cas9 expression levels were analyzed with an anti-Cas9 specific antibody (#Ab191468, AbCam, Cambridge, UK) by Western blot. The A673/Cas9 clones with constitutive expression of Cas9 were infected with lentiviral particles containing sgRNAs lentiviral expression vectors (see below) using an MOI of 1 or 5. Cells were selected with hygromycin (350 µg/mL) and cultivated for at least 50 days in the same conditions. Three different experiments with independent sgRNAs lentiviral infections were performed.

### 2.3. Selection of the Best Guide RNA (sgRNA) to Target EWSR1–FLI1

The A673 cells were infected with a commercial CRISPR library of 18,479 different sgRNAs targeting 1983 transcription factors including ten gRNAs located in the FLI1 gene (Supplementary Materials, Table S1) (#CB-11257, Thermo Fisher Scientific, Waltham, MA, USA). Two independent experiments were performed. In each experiment, a total of 150 × 10^6^ cells were infected at an MOI of 0.22 to obtain near 90% of cells infected with a unique sgRNA and a minimal representativeness of 500 cells per sgRNA. After 24 h, DNA was extracted for several plates and stored as control DNA. The remaining plates were then maintained in standard culture medium supplemented with puromycin (1 µg/mL) for 25 days, and then DNA was also extracted. Next, at least 27 µg of DNA from control and experimental conditions were PCR amplified with primers U6Primer-F (GGACTATCATATGCTTACCGTA) and Lenti-gRNA-R2 (TTCAAGTTGATAACGGACTAGC) which were located to both sides of each guide sequence. The PCR reactions were pooled, purified, indexed, and sequenced 1 × 150 in a NextSeq500 sequencer (Illumina, San Diego, CA, USA). At least 13 × 10^6^ reads were obtained per condition and experiment. The resulting Fastq files were processed in the web-based platform Galaxy [32] (relevant parameters are indicated in parentheses). First, Fastq files were trimmed with “Trim sequences” to extract the sequences exclusively corresponding to guide RNAs. Next, quality control was performed with FastQC, and reads with low average quality reads were filtered-out with “Trimmomatic” (AVGQUAL ≥ 25). Filtered-in reads were mapped against the guide sequences provided by the manufacturer using “BWA” (default parameters). Afterwards, “IdxStats” was used on BAM files generated in the previous step to quantify the number of reads for each guide (counts). Finally, data were exported to Excel spreadsheets and counts per each gRNA normalized by the total of counts in the experiment. This normalized value was compared between experimental and control conditions to calculate the enrichment score observed per gRNA. Selected gRNAs were cloned into lentiviral expression vectors to produce active sgRNAs in A673-infected cells (VectorBuilder, Chicago, IL, USA).

### 2.4. Cell Proliferation Assay

Cumulative population doubling was determined from cells maintained over 40–50 days in continuous growth. Cells were seeded at a density of 2.5 × 10^5^ cells in 100 mm plates. Before reaching confluence (80–90%), cells were trypsinized, and the number of viable cells was determined using trypan blue solution (NanoEnTek Inc., Seoul, Korea) and an EVE™ automatic cell counter (NanoEnTek Inc., Seoul, Korea). Afterwards, a fraction of the cells was seeded again at the same cell density, and the process was repeated until the end of the experiment. At several time points, the remaining cells were analyzed by flow cytometry or aliquoted, pelleted, and stored at −80 °C until DNA, RNA, or protein isolation was performed. The number of population doublings was calculated with the formula: n° of population doublings = log_2_ (numbers of cells at the initial time/number of cells at the final time). Cell doubling time was calculated in each cycle of cell seeding–trypsinization as cell doubling time = time elapsed between cycles/n° cell population doubling observed in this period of time.

### 2.5. DNA, RNA, and Protein Isolation

DNA, RNA, and total protein were isolated at different time points of the cell proliferation assay. At each time point, cells were trypsinized, washed with PBS, and distributed in three tubes. Pellets were stored at −80 °C until use. Genomic DNA was isolated using the QIAamp DNA Mini Kit (Qiagen, Hilden, Germany). RNA was extracted using TRI-REAGENT according to the manufacturer’s protocol (Sigma–Aldrich, Sant Louis, MI, USA) and additionally purified using RNeasy Mini Elute Cleanup kit (Qiagen, Hilden, Germany). Purified DNA and RNA were quantified using NanoDrop (Thermo Fisher Scientifics, Waltham, MA, USA). Total protein was isolated directly from cell pellets lysed in RIPA buffer (1× PBS, 0.1% SDS, 1% NP-40, 0.5% sodium deoxycholate) supplemented with a protease and phosphatase inhibitor cocktail (Roche, Basel, Switzerland). Protein concentration was determined with the Pierce BCA™ protein assay kit (Thermo Fisher Scientifics Waltham, MA, USA).

### 2.6. Confirmation of Gene Editing

DNA fragments covering the gRNA target were firstly amplified by PCR. Then, amplicons were analyzed by Sanger sequencing or next generation sequencing (NGS). The primer sequences used to amplify each region are described in Supplementary Materials Table S2. Sanger electropherograms were analyzed with the ICE CRISPR analysis tool (Synthego, Redwood City, CA, USA) to quantify, approximately, the percentage of indels mutations. For NGS, PCR products were indexed following the manufacturer’s instructions (New England Biolabs, Ipswich, MA, USA), purified, pooled, and sequenced in a MiSeq sequencer (Illumina, San Diego, CA, USA) using a 2 × 250 paired read setting. The average depth of coverage was 20,000× for each amplicon. The percentage of indels after Cas9-mediated editing was determined using Mosaic Finder, a bioinformatic software developed inhouse [33,34]. The Mosaic Finder pipeline integrates read mapping, normalization of read counts, mutation frequency calculation, and genome-editing efficiency statistics at each position of the target region. Briefly, Mosaic Finder takes the Fastq files generated by pair-end sequencing and generates consensus sequences by joining the corresponding read pairs (forward and reverse). The repertoire of consensus sequences (allelic clusters) represents the allelic diversity generated by the Cas9-mediated edition. These clusters are then aligned against the sequence used as reference and are classified in allelic classes based on the different type of the identified mutation (mismatch, insertion/deletion). The frequency of each cluster are then calculated and plotted.

### 2.7. Off-Target Analysis Using T7 Endonuclease I Assay

The BreakingCas tool was used to identify putative off targets for gRNA sequences [35], and gene editing at putative off-targets was analyzed using a T7 Endonuclease I assay that recognizes and cleaves non-perfectly matched DNA. PCR products obtained from genomic DNA were firstly purified using the QIAquick PCR Purification Kit (Qiagen, Hilden, Germany) and then 200 ng of the purified DNA was denatured for 5 min to 95 °C and afterwards allowed to align slowly using a first step consisting of a −2 °C/s ramp rate from 95 to 85 °C and a second step consisting of a −0.1 °C/s ramp rate from 85 to 25 °C. Next, 1 µL (10,000 UI/mL) of T7 Endonuclease I (#M0302, New England Biolabs, Ipswich, MA, USA) were added to the re-annealed PCR products and incubated at 37 °C for 15 min. The fragmented PCR products were analyzed by agarose electrophoresis, photographed, and the percentage of nuclease-specific cleavage products quantified by densitometry (Fiji software) [36]. 

### 2.8. Reverse Transcription and Quantitative PCR (RT-qPCR) 

A Taqman RT-qPCR assay was used to quantify the steady-state mRNA levels of EWSR1–FLI1, NR0B1, and TBP (used as a reference housekeeping gene) as described previously [37]. The CD44 mRNA steady-state levels were quantified using the SYBR-Green qPCR kit (Quantimix Easy Kit, #10606-4153, Biotools, Madrid, Spain). The PCR reactions were run on a Rotor Gene 6000 thermocycler (Qiagen, Hilden, Germany). The cycle threshold (Ct) for each gene and TBP was calculated using the RotorGene Software (v2.3.1). The relative expression for each gene was calculated as 2^−∆Ct^, where ∆Ct = Ct_gene_ − Ct_TBP_. Sequences of primers and TaqMan probes are shown in Supplementary Materials Table S3.

### 2.9. Western Blot 

For the Western blot, 10–20 µg of total protein extracts was subjected to electrophoresis on 4–15% polyacrylamide precast gels (Bio-Rad, Hercules, CA, USA) and blotted onto 0.2 µm PVDF membranes (Trans-Blot Turbo Mini, Bio-Rad, Hercules, CA, USA). Membranes were blocked with 5% skimmed milk powder in Tween-Tris Buffered Saline (TTBS) 1× and subsequently incubated with the corresponding primary antibodies diluted in the same buffer overnight at 4 °C. The next day, the membranes were washed in TTBS 1×, incubated with the secondary HRP-conjugated antibody, and washed again. The membranes were finally incubated with enhanced chemiluminescence (ECL) reactive (Merck Millipore, Darmstadt, Germany) and photographed on a ChemiDoc XRS+ System (BioRad, Hercules, CA, USA). Membranes were incubated sequentially with different antibodies after incubation with stripping buffer (Tris-HCL 1 M pH 6.8, SDS 10%, β-Mercaptoethanol 0.7%) for 30 min at 65 °C. The primary antibodies used were anti-FLI1 1:250 (#133485 Abcam, Cambridge, UK), anti-DAX1(NR0B1) 1:1000 (2F4 clone) [38], anti-CD44 1:1000 (#ab51037, Abcam, Cambridge, UK), and anti-β tubulin HRP 1:10,000 (#ab185057, Abcam, Cambridge, UK). Anti-mouse IgG (#sc-2055) and anti-rabbit IgG (#sc-2357) HRP-conjugated were purchased from Santa Cruz Biotechnology.

### 2.10. Immunocytochemical–Immunofluorescence (ICC/IF) Studies

The A673/Cas9/sgRNA and A673/sgRNA (control) cells were seeded on glass cover slides. After 72 h, the growth medium was removed, and the cells were washed in PBS. Then, cells were fixed and permeabilized for 15 min in ice-cold methanol. After, cells were incubated for 1 h in blocking solution (BS; 5% Goat Serum (*v*/*v*) in PBS 1×), washed with PBS twice, and incubated overnight at 4 °C with anti-FLI1 rabbit monoclonal antibody (1:700 in BS; ab133485, AbCam, Cambridge, UK). The next day, cells were washed 4 × 10 min with PBS and incubated for 1.5 h at room temperature with the secondary antibody (1:500; Goat anti-rabbit IgG-Alexa Fluor 594, Abcam, Cambridge, UK) supplemented with 4% FBS. Cells were counterstained for 4 min with the nuclear stain 4,6-diamidino-2-phenylindole (DAPI), washed 4 × 10 min with PBS, and mounted on slides using ProLong™ Gold antifade mounting medium (#P36934, Thermo Fisher Scientific, Waltham, MA, USA). Cells were visualized in a fluorescence microscope (Leica, Wetzlar, Germany).

### 2.11. Cell Cycle Analysis by Flow Cytometry 

Around one million cells were trypsinized, centrifugated, and resuspended in 400 µL of PBS 1×. Then absolute ethanol was added for at least 1 h at 4 °C to fix cells. Next, cells were washed with PBS 1× and centrifuged. The pellet was incubated at 37 °C for 30 min in darkness in a solution containing 50 µg/mL propidium iodide and 100 µg/mL RNAase A in PBS 1×. Cells were analyzed in a MACS Quant Analyzer 10 flow cytometer (Miltenyi Biotec, Cologne, Germany), and the results were analyzed using FlowJo™ software (v.10.7.1, De Novo Software, Pasadena, CA, USA). 

### 2.12. Senescence Assay

To assess the senescence state of cells, β-galactosidase activity, a known characteristic of senescent cells, was measured. The Senescence-β-Gal Staining Kit (Cell Signaling Technology, Danver, MA, USA) was used according to the manufacturer’s protocol. Briefly, cells were seeded by duplicate in a 6-multiwell plate to a density of 40,000–50,000 cells per well for 48 h. Then, cells were washed with PBS 1× and fixed with a fixative solution for 10–15 min at room temperature and washed with PBS 1× twice. Then, 1 mL of β-Galactosidase Staining Solution, containing 1 mg/mL X-Gal, was added per well and the plate incubated at 37 °C, for 24–48 h in a dry incubator. A total of 20 fields were photographed per experimental condition under a microscope and β-galactosidase-positive cells counted by two independent researchers and the percentage of positive cells versus total cells calculated.

## 3. Results

### 3.1. Selection of Guide RNAs Targeting EWSR1–FLI1

**Figure 1:**
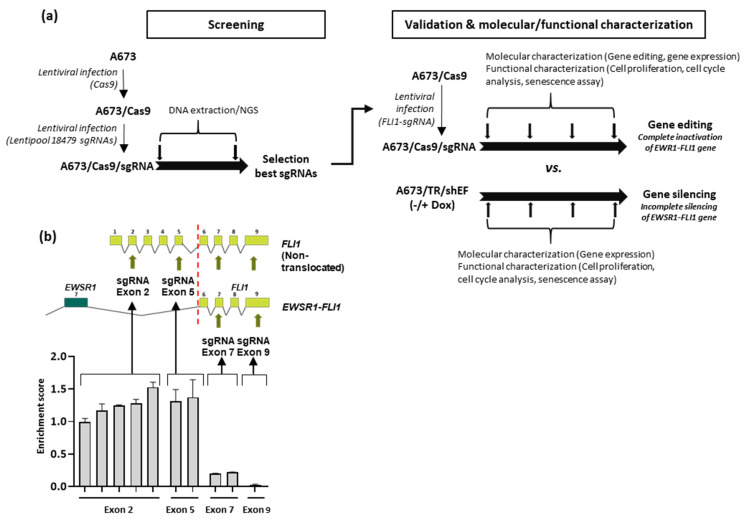
Selection of gRNAs targeting EWSR1–FLI1 and analysis of gene editing evolution. (**a**) Experimental design: Ewing sarcoma cell line A673 expressing Cas9 protein (A673/Cas9) were generated by lentiviral infection. After clonal selection, A673/Cas9 cells were infected with a multiplex lentiviral CRISPR library of 18,479 different sgRNAs targeting 1983 transcription factors including ten gRNAs targeting FLI1. After this screening phase, two gRNAs were selected for functional and molecular characterization. A673/Cas9 were infected with lentiviral sgRNAs to generate A673/Cas9/sgRNA cells and then maintained in continuous growth to assess gene editing, cell proliferation, senescence, and studies of mRNA and protein expression at different time points. A673/TR/shEF, which expresses a specific EWSR1–FLI1 shRNA upon doxycycline stimulation, were cultured and analyzed in a similar way. The results obtained upon gene editing and gene silencing were then compared. (**b**) Schematic representation of native FLI1 and EWSR1–FLI1 fusion genes, location of FLI1 gRNAs, and enrichment scores obtained for each gRNA in the CRISPR screening assay (mean ± SD of two independent experiments).

We chose the cell line A673 as a model to analyze the effect of EWSR1–FLI1 gene inactivation in Ewing sarcoma cells, since this cell line is probably the Ewing sarcoma cell line more broadly used to study Ewing sarcoma pathogenesis, particularly with regard to the mechanism of EWSR1–FLI1-mediated oncogenesis. To select specific gRNAs targeting the EWSR1–FLI1 gene, we took advantage of the data generated from a multiplex CRISPR screening assay targeting 1983 transcription factors (Figure 1a). This library includes ten different gRNAs targeting four different exons of FLI1. We first infected A673 cells with lentivirus containing a vector encoding Cas9 to generate a stable cell line (A673/Cas9) (Supplementary Materials, Figure S1) and afterwards, we infected the A673/Cas9 cells with the CRISPR library targeting the transcription factors. Cells were cultured in selection medium for 25 days, and the enrichment scores for each gRNAs were obtained as described in detail in Materials and Methods (Section 2). As shown in Figure 1b, the enrichment scores for each gRNA targeting FLI1 were largely dependent on the location of the target sequence. Thus, gRNAs targeting FLI1 exons 2 and 5 had enrichment scores near one or greater, indicating that targeting these exons did not have an effect on cell viability. This agrees with the fact that the FLI1 wild type is not expressed in Ewing sarcoma cells [12] and, therefore, targeting the exons of FLI1 that are not included in the EWSR1–FLI1 fusion protein are expected to have no effect. However, gRNAs targeting exons 7 and 9 of FLI1, which are included in the chimeric protein, showed a significant reduction in the enrichment score values, confirming a deleterious effect in the A673 cells (Figure 1b). In view of this data, we chose two gRNAs for experimental validation and additional molecular and functional characterization. One of them was the gRNA located in exon 9, which had the most severe effect on cell proliferation. The other, which was one of the gRNAs located in exon 2, did not affect cell viability and was used here as a negative control (Supplementary Materials, Table S1). 

Both gRNAs were cloned into lentiviral vectors under the control of a U6 promoter and afterwards used to infect A673/Cas9 cells. Then, cells were maintained in selection medium over 40–50 days to assess the effect of EWSR1–FLI1 gene inactivation over time (Supplementary Materials, Figures S2–S5). Cell proliferation assays, cell cycle analysis, senescence analysis, and mRNA and protein expression studies were performed at different time points (Figure 1a). The results were compared with those obtained in A673/TR/shEF cells, a well-established EWSR1–FLI1 silencing model based on a doxycycline-inducible shRNA system (A673/TR/shEF) [12]. This allowed us to compare the effects of the EWSR1–FLI1 gene editing versus EWSR1–FLI1 gene silencing (Figure 1a). 

### 3.2. Effect of EWSR1–FLI1 Gene Editing on EWSR1–FLI1 Expression and EWSR1–FLI1 Targets

Once gene editing on EWSR1–FLI1 was confirmed, we decided to assess its effect on EWSR1–FLI1 expression as well as on two surrogate markers of EWSR1–FLI1 activity: NR0B1 (DAX1), a gene regulated positively by EWSR1–FLI1 [11], and CD44, a gene regulated negatively by EWSR1–FLI1 [39] (unpublished results). We compared the effects observed in CRISPR/Cas9 edited cells with those observed in A673/TR/shEF upon doxycycline stimulation (gene silencing). 

**Figure 2:**
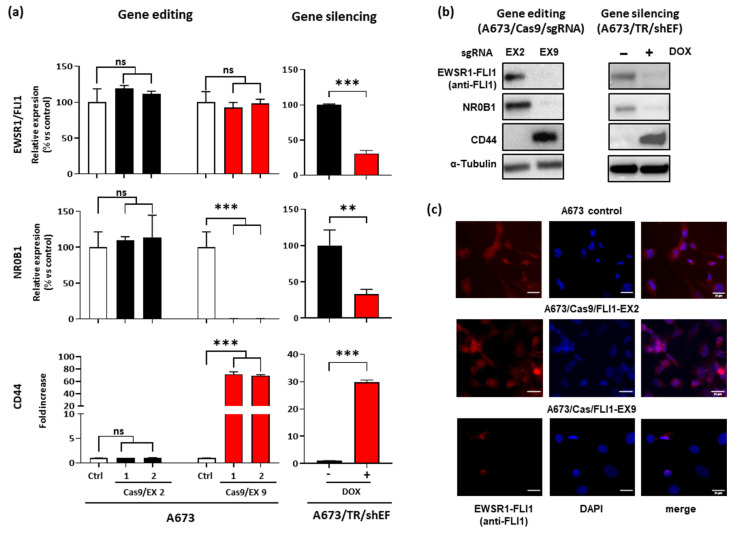
Effect of EWSR1–FLI1 gene editing on EWSR1–FLI1 expression and EWSR1–FLI1 target genes. (**a**) EWSR1–FLI1, NR0B1, and CD44 mRNA levels were quantified by RT-qPCR in A673/Cas9 cell lines infected with sgRNA vectors targeting exon 2 and exon 9. The graphs represent the results of two independent experiments performed with MOI = 1 (1) and MOI = 5 (2). A673/sgRNA was used as the control (Ctrl). A673/TR/shEF cells were stimulated with doxycycline (1 µg/mL) for 72 h to induce the expression of the EWSR1–FLI1-specific shRNA. Data shown are the mean ± SD of experiments conducted in triplicate (ns, not significant; ** *p* < 0.01; *** *p* < 0.001; Student’s *t*-test). (**b**) EWSR1–FLI1, NR0B1, and CD44 protein levels were detected by Western blot. β-Tubulin was used as a control for loading and transferring. (**c**) Immunostaining of cells with anti-FLI1 antibody. Intense nuclear staining for EWSR1–FLI1 (red fluorescence colocalized with DAPI staining) was observed in both the A673 control cell line and A673/Cas9/FLI1-EX2, while it was undetectable in the majority of the A673/Cas9/FLI1-EX9 cells.

As shown in Figure 2a, gene editing on FLI1 exon 2 had a negligible effect on EWSR1–FLI1 mRNA levels. Interestingly, gene editing on FLI1 exon 9 also had no effect on EWSR1–FLI1 mRNA levels. This suggests that although gene editing was verified by sequencing (Figures S2 and S3), it did not seem to affect the steady-state levels of EWSR1–FLI1 mRNA. As previously reported, the stimulation of A673/TR/shEF cells with doxycycline reduced the levels of EWSR1–FLI1 mRNA nearly 70% [11]. Figure 2a also shows the results regarding EWSR1–FLI1 gene targets NR0B1 and CD44. Gene editing on FLI1 exon 2 had no effect on NR0B1 and CD44 mRNA levels, suggesting that EWSR1–FLI1 was not affected in these cells. However, gene editing in exon 9 reduced the levels of NR0B1 dramatically until almost undetectable levels and increased the levels of CD44 more than 60-fold, indicating that although steady-state levels of EWSR1–FLI1 mRNA were unaltered, EWSR1–FLI1 protein was probably not produced or it was not functional. As expected, NR0B1 and CD44 mRNA levels decreased and increased respectively upon EWSR1–FLI1 silencing in A673/TR/shEF cells. In the case of NR0B1, the effect of gene editing on mRNA levels was clearly more intense than that observed upon gene silencing.

To confirm the effect of gene editing at the protein level, we analyzed the levels of EWSR1–FLI1, NR0B1, and CD44 by Western blot. As shown in Figure 2b, EWSR1–FLI1 and NR0B1 protein levels were reduced until nearly undetectable levels in A673/Cas9/FLI1-EX9 cells when compared to A673/TR/Cas9/FLI1-EX2 cells. In agreement with mRNA levels, CD44 protein was upregulated in A673/Cas9/FLI1-EX9. Similar results were obtained in A673/TR/shEF stimulated with doxycycline compared to unstimulated control cells.

Finally, we analyzed the expression of EWSR1–FLI1 by immunofluorescence in cell cultures (Figure 2c). EWSR1–FLI1 was detected in the nucleus of control and A673/Cas9/FLI1-EX2 cells but was undetectable in the majority of A673/Cas9/FLI1-EX9 cells. Taken together, these results demonstrate that gene editing of FLI1 at exon 9 was able to efficiently knockout the EWSR1–FLI1 gene and, consequently, the expression of the corresponding protein.

### 3.3. Effect of EWSR1–FLI1 Gene Inactivation on Cell Proliferation

**Figure 3:**
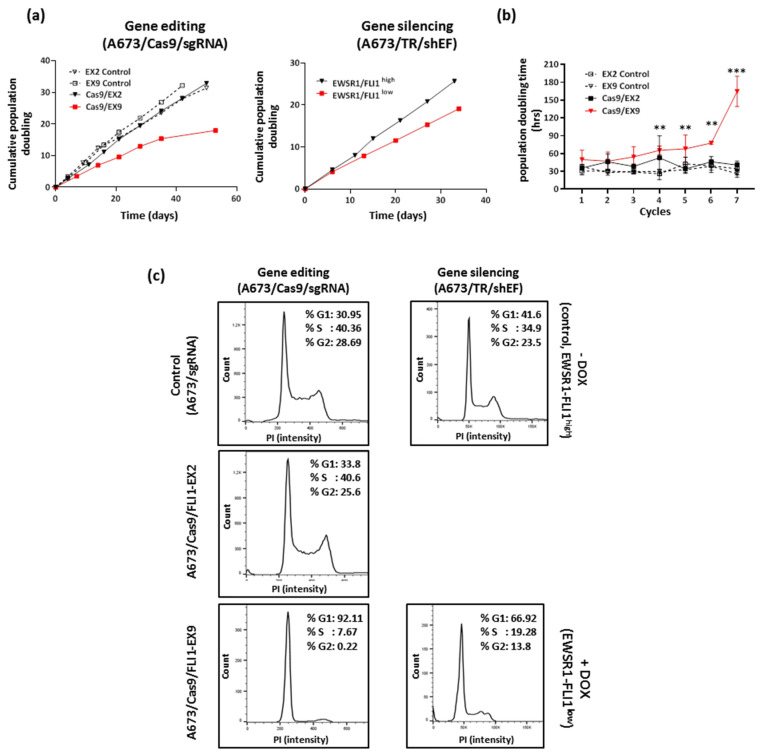
Effect of EWSR1–FLI1 gene editing on cell proliferation. (**a**) A673/Cas9/sgRNA cells were continuously maintained in culture and the cumulative population doubling was recorded. The A673/TR/shEF cells were also continuously maintained in culture in the absence or presence of doxycycline (1 µg/mL). (**b**) Quantification of the population doubling time in each cycle of cell seeding–trypsinization. Mean ± SD of three independent experiments is shown (ns, not significant; ** *p* < 0.01, *** *p* < 0.001; two-way ANOVA Tukey’s post hoc vs. control). (**c**) The cell cycle was analyzed by flow cytometry in non-synchronized cells. The percentages of cells in each cell cycle phase are indicated. One representative experiment out of two independent experiments performed with similar results is shown.

Next, we analyzed the effect of EWSR1–FLI1 gene editing on cell proliferation by quantification of the cumulative population doubling for 45–55 days (Figure 3a). Gene editing on the FLI1 exon 9 (A673/Cas9/FLI1-EX9) resulted in a significant reduction in proliferation compared to control cells (non-edited). By contrast, no effects on cell proliferation were observed in the A673 cells edited on FLI1 exon 2 (A673/Cas9/FLI1-EX2) compared to the control. Figure 3b shows the average of the population doubling time calculated from three independent experiments over time. The cell doubling time at the end of the experiment increased significantly in A673/Cas9/FLI1-EX9 cells (164.6 ± 25.0 h) in comparison to the control or A673/Cas9/FLI1-EX2 cells (40.7 ± 6.8 h; *p* < 0001, two-way ANOVA, Tukey’s post hoc). Interestingly, the effect of EWSR1–FLI1 gene editing in A673/Cas9/FLI1-EX9 cells on cell proliferation was greater than that observed upon EWSR1–FLI1 knockdown in A673/TR/shEF cells. As shown in Figure 3a, continuous stimulation of these cells with doxycycline over 35 days significantly reduced the cumulative population doubling and increased the cell doubling time but to a lesser extent than that observed in A673/Cas9/FLI1-EX9. These results suggest that inactivation of EWSR1–FLI1 by gene editing was able to affect proliferation in a more severe way than EWSR1–FLI1 silencing.

The evolution of gene editing (Figure S3) correlated with the proliferation profile observed. Thus, the higher percentages of gene editing on exon 9 observed at the end of the experiment were associated with the higher cell doubling time. This fact is compatible with a strong addiction of Ewing sarcoma cells to EWSR1–FLI1, an essential gene for these cells and whose gene editing and subsequent inactivation result in blockage of cell proliferation.

To confirm the effect of EWSR1–FLI1 gene editing on cell proliferation, we analyzed the different phases of cell cycle by flow cytometry. As shown in Figure 3c, A673/Cas9/FLI1-EX9 cells showed a near-complete blockage of the cell cycle as evidenced by the fact that more than 90% of cells were in the G1 phase. EWSR-FLI1 silencing in A673/TR/shEF also produced a significant increase in the percentage of cells in the G1 phase (nearly 67%) in comparison to control cells (42%) but to a lesser extent than that observed in A673/Cas9/FLI1-EX9. Taken together, these results demonstrate that EWSR1–FLI1 gene inactivation by gene editing produces a practically complete blockage of cell proliferation.

### 3.4. EWSR1–FLI1 Inactivation Produces Generalized Senescence

**Figure 4:**
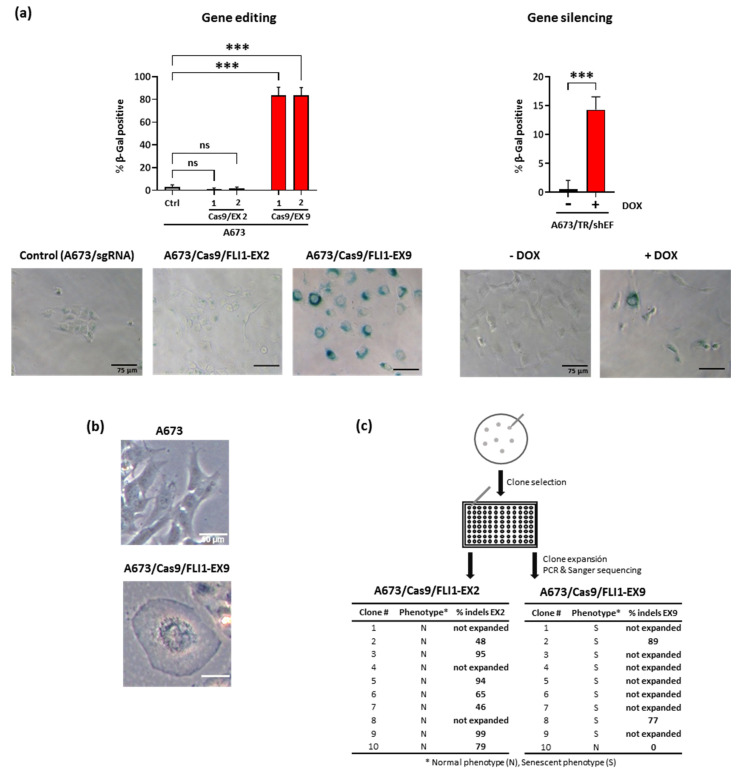
EWSR1–FLI1 gene inactivation induced generalized senescence. (**a**) β-Galactosidase activity was measured in control (Ctrl) cells (A673/sgRNA) and A673/Cas9/sgRNA cells using a β-galactosidase activity assay. β-Galactosidase-positive cells were counted, and the percentage of positive cells were determined. The graphs represent the results of two independent experiments performed with MOI = 1 (1) and MOI = 5 (2). The A673/TR/shEF cells were stimulated with doxycycline (1 µg/mL) for 7 days to induce the expression of the EWSR1–FLI1-specific shRNA (mean ± Scheme 0. Student’s *t*-test). Representative micrographs of each cell line are also shown. (**b**) Representative micrographs of A673 and A673/Cas9/FLI1-EX9 cells, showing the characteristic appearance of senescent cells in A673/Cas9/FLI1–EX9 cells. (**c**) The A673/Cas9/sgRNAs cells were seeded at a low density and then isolated clones were reseeded independently in 96-well plates. Afterward, Sanger sequencing/ICE CRISPR analysis was performed for each clone to determine the gene edition percentage (% indels). The tables show the phenotype of each picked-up clone, if it expand after reseeding and the percentage of gene edition.

The severe cell cycle arrest observed in A673/Cas9/FLI1-EX9 cells is compatible with a senescence phenotype. Thus, we analyzed whether the EWSR1–FLI1 gene inactivation produced a senescent state in A673 cells. With that purpose, we analyzed the β-galactosidase activity, an accepted surrogate marker of senescence [40]. As shown in Figure 4a, more than 80% of A673/Cas9/FLI1-EX9 cells stained positively for β-galactosidase activity. By contrast, less than 5% of cells were β-galactosidase positive in the control (A673/sgRNA) and A673/Cas9/FLI1-EX2. The A673/Cas9/FLI1-EX9 cells displayed the typical morphological characteristics of senescent cells such as a flattened aspect and a vacuolized cytoplasm (Figure 4b). EWSR1–FLI1 knockdown in A673/TR/shEF also induced a senescent phenotype, although at a much lower level in comparison to Cas9 edited cells on FLI1 exon 9 (Figure 4a). 

To further characterize this phenotype, A673/Cas9/sgRNAs cells were seeded at low density and cultured for 15 days to allow for the formation of colonies (Figure 4c). Afterwards, colonies were selected based on their morphological appearance (senescent vs. normal phenotype), picked-up, reseeded in 96-well plates, and cultured for an additional 15 days until the colony grew large enough to be able to extract DNA. DNA was afterwards analyzed for gene editing using Sanger sequencing and the ICE CRISPR tool. All colonies of A673/Cas9/FLI1-EX2 cells showed the characteristic phenotype of normal A673 cells in culture. Out of ten selected colonies, seven expanded after reseeding, and all of them showed gene editing in exon 2 of FLI1. By contrast, two types of colonies were clearly recognizable in A673/Cas9/FLI1-EX9 cells; a few colonies had the typical appearance of A673 cells, while the majority of the colonies displayed the aspect of senescent cells. The only normal colony that was picked out expanded quickly after reseeding. Interestingly, this colony showed no gene editing at all in FLI1 exon 9, which is compatible with a wild-type EWSR1–FLI1 gene. In contrast, only two out of nine colonies with a senescent phenotype grew after replanting. In this case, both colonies showed gene editing in exon 9 of FLI1. These results suggest that senescent colonies had more problems expanding after trypsinization, confirming the deleterious effect caused by the genetic inactivation of EWSR1–FLI1.

## 4. Discussion

Despite international collaborative efforts to improve Ewing sarcoma’s outcome, the progress made over the last decades has been rather limited [2,3,4]. High-intensity chemotherapy regimens are the standard treatment for these patients, and although this approach is effective and even curative in a significant proportion of patients with localized tumors, the prognostic of patients with metastatic disease or late relapses is very poor, with 5 year overall survival figures below twenty five percent [5]. There is, therefore, a consensus in the field that it is absolutely indispensable to identify new innovative strategies that can represent a real advance in the disease’s treatment. These advances can only come by focusing on the tumor biology. 

The molecular hallmark of Ewing sarcoma is the presence of chromosomal translocations that give rise to chimeric transcriptional factors that act as potent oncogenes governing the transformation process. The fact that these fusion proteins, such as EWSR1–FLI1, are the initial event that trigger the oncogenic events, makes this tumor ideal for the development of targeted therapies [19]. With this idea in mind, several groups have attempted to develop drugs able to interfere with the transcriptional activity of EWSR1–FLI1 fusion proteins. Among these drugs are trabectedin and its analogs [41,42], Mithramycin [43,44], and YK-4–279 and derivatives [45,46]. Although these drugs have reached clinical testing, the results obtained have been, at the moment, discouraging because of the elevated toxicity in some cases [47] and for the intrinsic difficulty in targeting transcription factors with pharmacological approaches [48], not to mention that pharmacological inhibition of EWSR1–FLI1 could require the administration of a drug for prolonged periods to achieve a durable response, as occurs in the case of tyrosine kinase inhibitors (for example, imatibib) [49]. This could lead to a decrease in the drug’s efficacy or the appearance of resistance in the clinical setting [50]. Therefore, to obtain the best possible results, it would be desirable to obtain a complete and long-lasting inactivation of EWSR1–FLI1.

One way to obtain the inactivation of a determined gene could be the use of gene therapy strategies. Complete gene inactivation through a genetic approach was unthinkable only a few years ago, as we did not have the proper tools to conduct it. However, thanks to the discovery of the CRISPR/Cas9 system, new opportunities have emerged in gene therapy applied to cancer [22,51]. Although gene therapy-based approaches for treating cancer are still in their early stages, and they are not without hurdles, there is great interest from academia and industry in the development of these new approaches, which indicates that these therapies can be a reality in the medium and long term [26,51]. 

Effective gene therapy for cancer must address at least three critical issues. First, it must pursue the inactivation of essential genes for tumor growth, both at the primary site and, more importantly, at metastatic sites. Second, it must be specific for the gene(s) of interest. Third, it must include a mechanism that allows for transportation of the gene editing machinery. In this work, we aimed to answer the first two questions in the context of Ewing sarcoma. The next step will therefore be to develop a type of nanotherapy that allows for transporting the gene editing machinery specifically to Ewing sarcoma cells, for which it will be necessary not only to develop the vehicle itself but to design the elements that allow the specific identification of target cells. The development of this methodology will make it possible to analyze the efficacy of this therapy in preclinical models (e.g., growth of primary xenografts and metastasis formation) and, thus, determine the real viability and efficacy of this gene therapy.

We have shown that gene inactivation of EWSR1–FLI1 using a CRISPR/Cas9 gene editing approach resulted in a highly efficient method to utterly knockout EWSR1–FLI1 in the Ewing sarcoma cell model A673. To our knowledge, no complete inactivation of EWSR1–FLI1 in Ewing sarcoma cells has been reported until now. In addition, our results confirm and extend the finding that Ewing sarcoma cells are “addicted” to EWSR1–FLI1, ratifying the fact that targeting EWSR1–FLI1 is an excellent approach for cancer gene therapy.

We have shown that a gRNA targeting exon 9 of FLI1, selected from a screening performed with a multiplex CRISPR library, is able to edit EWSR1–FLI1 efficiently. Gene editing at exon 9 of FLI1 reduced the expression of the EWSR1–FLI1 protein until undetectable levels and altered the expression of two well-documented EWSR1–FLI1 gene targets (the EWSR1–FLI1-upregulated gene NR0B1 and the EWSR1–FLI1-downregulated gene CD44). Our study in this regard is limited but suggests that total inactivation of EWSR1–FLI1 by gene editing strengthens the expression profile induced upon EWSR1–FLI1 knockdown using shRNA strategies. Further experiments including RNAseq, ChIP-seq, or 3C methodologies, as carried out in other studies [9,52,53], would allow deeper insight into the dysregulated expression patterns following complete inactivation of EWSR1–FLI1. 

More interestingly, EWSR1–FLI1 gene editing at exon 9 of FLI1 had a dramatic effect on cell proliferation. Thus, gene editing totally blocked cell proliferation by resting the cells in the G1 phase, associated with a generalized induction of cellular senescence. We compared the effect of gene editing on protein expression, cell proliferation, and induction of senescence with those produced in a well-established model of EWSR1–FLI1 silencing mediated by shRNAs (A673/TR/shEF cells). In all the parameters analyzed, gene editing was significantly more effective than gene silencing, but the differences were especially striking with regard to the inhibition of cell proliferation and induction of cellular senescence. Senescence is a cellular state and the induction of its mechanisms are not entirely well understood. In cancer cells, certain combinations of oncogenes, tumor suppressor genes, hypoxia, stress, etc., can produce senescence. Even certain stimuli can produce senescence in a certain tumor cells depending on the presence or absence of determined mutations [54,55]. The exact mechanism that causes senescence in A673 cells upon EWSR1–FLI1 inactivation are unknown; thus, the characterization of these mechanisms can provide new insights into the pathogenesis of Ewing sarcoma. In this work, we used β-galactosidase staining to identify senescent cells. Although this is one of the most used techniques, it presents some limitations since β-galactosidase can also accumulate in certain experimental situations such as high-density culture or serum starvation [56,57,58,59]. However, since none of these circumstances were present in our experiments (cells were grown under standard serum conditions (10%) and were never allowed to reach confluence), we consider that the β-galactosidase staining assays faithfully reflect the existence of true senescent cells. 

According to our screening assay, only gRNAs targeting FLI1 exons involved in the EWSR1–FLI1 gene showed an effect on cell viability. While these results were expected, the screening also showed that gRNA targeting exon 9 of FLI1 was more effective than those targeting exon 7. Although in silico approaches can potentially identify many factors that can affect the gene editing efficiency [60,61], our results highlight the need to experimentally validate different gRNAs to identify those that produce the highest levels of efficiency in terms of gene editing and, therefore, more relevant from a functional point of view. Our results also suggest that the selected gRNA had minimal, if any, off-target effects. Although the frequency of off-targets was initially thought to be high, several recent studies have shown that the occurrence of off-targets is actually low [62,63]. In fact, analysis of two possible off-targets did not show any evidence of editing (Supplementary Materials, Figure S5). Interestingly, one of these putative off-target genes corresponded to ERG, another member of the ETS family of transcription factors involved in chromosomal translocations in Ewing sarcomas [1]. Therefore, it is very remarkable that although FLI1 and ERG are closely related genes, the selected FLI1 gRNA was able to discriminate between both genes and only produce gene editing in FLI1.

Recently, it has been proposed that EWSR1–FLI1 levels in Ewing sarcoma cells can fluctuate between a state of high expression (EWSR1–FLI1^high^) and a low expression one (EWSR1–FLI1^low^) [53,64]. According to this hypothesis, EWSR1–FLI1^high^ cells would have a high proliferative index but a low capacity to disseminate. By contrast, EWSR1–FLI1^low^ cells would have a low proliferative index but a high capacity to disseminate. These EWSR1–FLI1^low^ cells would therefore be resistant to chemotherapy and potentially responsible for the late relapses that so frequently occur in Ewing sarcoma, usually with fatal results. Although this hypothesis could be attractive, it must be clearly demonstrated experimentally. In any case, this hypothesis may have important implications for therapies that are aimed at inactivating the activity of EWSR1–FLI1, particularly if this inactivation is only partial as is expected from pharmacological approaches, since although such drugs were especially selective and specific, it is unlikely that they could completely block the transcriptional activity of EWSR1–FLI1. If the inactivation of EWSR1–FLI1 is not complete, then the use of these drugs could increase EWSR1–FLI1^low^ cells, thus favoring the population of cells with a propensity to metastasize. This possible problem could be avoided if, instead of partial inactivation, a total inactivation of EWSR1–FLI1 is achieved as that observed after EWSR1–FLI1 gene editing. In light of our results, we hypothesized that complete inactivation of EWSR1–FLI1 in vivo would result in EWSR1–FLI1^null^ senescent cells that would ultimately be cleared in vivo by tissue homeostasis [65] or pharmacological elimination [66]. Interestingly, senescent cells are capable of secreting a set of substances (the so-called senescent secretome) that, in turn, can produce senescence in neighboring cells [54,55,67]. We speculate that the elimination of EWSR1–FLI1 in a limited group of tumor cells could reduce the proliferation of neighboring cells through the secretion of pro-senescent factors, thus amplifying the effect of a possible therapy aimed at permanently inactivating EWSR1–FLI1.

In summary, we described a gene therapy approach to targeting the EWSR1–FLI1 oncogene characteristic of Ewing sarcoma. We identified a gRNA sequence that showed a high level of editing efficiency and specificity, producing full cycle arrest and generalized cellular senescence. Although this strategy could be useful to target the 85% of Ewing sarcomas harboring the EWSR1–FLI1 fusion gene, a different approach should be developed to target Ewing sarcomas with other gene fusions involving other ETS genes, such as EWSR1-ERG (10% of cases), and even other rarer gene fusions such as EWSR1-ETV1, EWSR1-ETV4, or EWSR1-FEV (5% of cases). A similar experimental strategy, combining a pre-screening phase with a validation phase aimed at functionally validating the best sgRNAs targeting these ETS genes, should provide the basic elements for developing a gene editing therapy able to inactivate the fusion genes less frequent in Ewing sarcoma.

As with any other anti-cancer therapy, an anti-cancer gene therapy is not without side effects. In this regard, targeting exon 9 of FLI1 could have consequences for several cells of the hematopoietic system in which the FLI1 transcription factor plays a key role in the differentiation of these cells [68]. Consequently, the next step on the long road to a future clinical application of gene therapy in Ewing sarcoma is the development of a safe strategy to transport the gene-editing machinery to the tumor cells. Although this is the most complex point of the experimental development, the high level of research activity that is taking place in this field allows us to have well-founded hopes for the future [69,70,71,72].

## Data Availability

The data presented in this study are available from the corresponding author on reasonable request.

## Supplementary Materials

The following are available online at https://www.mdpi.com/article/10.3390/cancers13153783/s1, Figure S1: Establishment of A673/Cas9 cells; Figure S2: Confirmation of gene editing at the specified locus in A673/Cas9 cells infected with sgRNAs; Figure S3: Quantification of gene editing rate at different time points; Figure S4: Detailed analysis of Cas-9 mediated gene editing; Figure S5: Off-target analysis; Figure S6: Original Western blots of Figure 2b; Table S1: gRNAs targeting FLI1; Table S2: Primers used for gene editing analysis by sequencing and T7 endonuclease I mismatch assay; Table S3: Primers used in quantitative PCR assays.
