# Supplementary material for: Therapeutic Potential of EWSR1–FLI1 Inactivation by CRISPR/Cas9 in Ewing Sarcoma
Source: Cancers (Basel). 2021 Jul 27;13(15):3783. doi: 10.3390/cancers13153783 (PMC8345183; doi:10.3390/cancers13153783)
Supplement: Supplementary file 1 [file cancers-13-03783-s001.zip › 210325_EWSKO_Supplementary_Tables_DEF.pdf]

Cervera et al.

Supplementary tables

Table S1. gRNAs sequences targeting FLI1 included in the CRISPR library and enrichment scores obtained in two independent experiments. gRNA target selected for additional molecular and functional characterization are shaded.

Target sequence	PAM sequence	Genomic position (1)	ES Exp1 (2)	ES Exp2 (2)
TCCCTCTTTGACTCAGCGTA	CGG	128758145(+)(Ex2)	0.953	1.033
TCCGTACGCTGAGTCAAAGA	GGG	128758149(-)(Ex2)	1.241	1.103
CTCCGTACGCTGAGTCAAAG	AGG	128758150(-)(Ex2)	1.245	1.255
CTTTGACTCAGCGTACGGAG	CGG	128758150(+)(Ex2)	1.233	1.322
CGGGAGTATGACCACATGAA	TGG	128758298(+)(Ex2)	1.473	1.584
TCGTGAGGATTGGTCGGTGT	GGG	128781988(-)(Ex5)	1.440	1.185
ACTCAATCGTGAGGATTGGT	CGG	128781994(-)(Ex5)	1.565	1.185
GATCGTTTGTGCCCCTCCAA	GGG	128807189(-)(Ex7)	0.192	0.200
TGATCGTTTGTGCCCCTCCA	AGG	128807190(-)(Ex7)	0.221	0.217
CAAAATGACGGACCCCGATG	AGG	128810556(+)(Ex9)	0.017	0.033

[1] Nucleotide position according hg38 consensus genome. In brackets it is indicated orientation (+/-) and exon number. (2) ES: Enrichment score obtained in each experiment

Table S2. Primers used for gene editing analysis by sequencing (Sanger sequencing or next generation sequencing (NGS)) and T7 endonuclease I mismatch assay (T7EI)

Primer	Sanger/NGS/ T7EI	Sequence
FLI1-EX2-F	Sanger/NGS	5′-TGTCACTTGCTTGGGTGAAG-3′
FLI1-EX2-R	Sanger/NGS	5′-GTTGACCCTCACTGGCTGAT-3′
FLI1-EX2-F	T7EI	5′-CCACTATTCTTGGCCTCCCT-3′
FLI1-EX2-R	T7EI	5′-TTGACCCTCACTGGCTGATT-3′
FLI1-EX9-F	Sanger/NGS	5′-TCTCTGGGCTGAGGTGTTCT-3′
FLI1-EX9-R	Sanger/NGS	5′-ATTCATGTTGGGCTTGCTTT-3′
FLI1-EX9-F	T7EI	5′-TTCTCTCCCGTTTGCCTCAC-3′
FLI1-EX9-R	T7EI	5′-TGTGATGCGGCTCCAAAGAA-3′
AC244230.2-F (Off target gRNA Ex2)	T7EI	5′-TGGCCCTTATCTAATAGCGCA-3′
AC244230.2-R (Off target gRNA Ex2)	T7EI	5′-TATCGCACTGTGGTCCAAGA-3′
PTK2-F (Off target gRNA Ex2)	T7EI	5′-AATGGAGGATGTGGTGTGGG-3′
PTK2-R (Off target gRNA Ex2)	T7EI	5′-CTGTGCCCTGCCAAATGTAT-3′
Acot11-F (Off target gRNA Ex9)	T7EI	5′-CCTGCTGCTTGGACAACAT-3′
Acot11-R (Off target gRNA Ex9)	T7EI	5′-TGCTGGCAGTGGTAGCTCTT-3′
ERG-F (Off target gRNA Ex9)	T7EI	5′-CATGTACGGGAGGTCTGAGG-3′
ERG-R (Off target gRNA Ex9)	T7EI	5′-CAGATCCAGCTTTGGCAGTT-3′

Table S3. Primers used in quantitative PCR assays.

Primer	Taqman / SYBR Green	Sequence
EWSR1-FLI1-F	Taqman	5′-AGCCAAGCTCCAAGTCAATATAG-3′
EWSR1-FLI1-R	Taqman	5′-TCCTCTTCTGACTGAGTCATAAG-3′
EWSR1-FLI1-probe	Taqman	5′-(FAM)-AACAGAGCAGCAGCTACGGGCAGCA-(BHQ1)-3′
NR0B1-F	Taqman	5′-GAATGTACTTCACGCACTGCAGG-3′
NR0B1-R	Taqman	5′-TTTCTTTCCAAATGCTGGAGTCTGA-3′
NR0B1-probe	Taqman	5′-(FAM)-CATCAGTACCAAGGAGTACGCCTACCT-(BHQ1)-3′
CD44-F	SYBR Green	5′-TGGAGGACAGAAAGCCAAGT-3′
CD44-R	SYBR Green	5′-CCACATTCTGCAGGTTCCTT-3′
TBP-F	Taqman	5′-GAACATCATGGATCAGAACAACAG-3′
TBP-R	Taqman	5′-ATTGGTGTTCTGAATAGGCTGTG-3′
TBP-probe	Taqman	5′-(HEX)-CTGCCACCTTACGCTCAGGGCTTHH-(BHQ1)-3′
