# Supplementary material for: Therapeutic Potential of EWSR1–FLI1 Inactivation by CRISPR/Cas9 in Ewing Sarcoma
Source: Cancers (Basel). 2021 Jul 27;13(15):3783. doi: 10.3390/cancers13153783 (PMC8345183; doi:10.3390/cancers13153783)
Supplement: Supplementary file 1 [file cancers-13-03783-s001.zip › 210712_EWSKO_Supplementary_Figures_2nd_revision_v1_JA_RM_DEF.pdf]

Supplementary figures

Figure S1

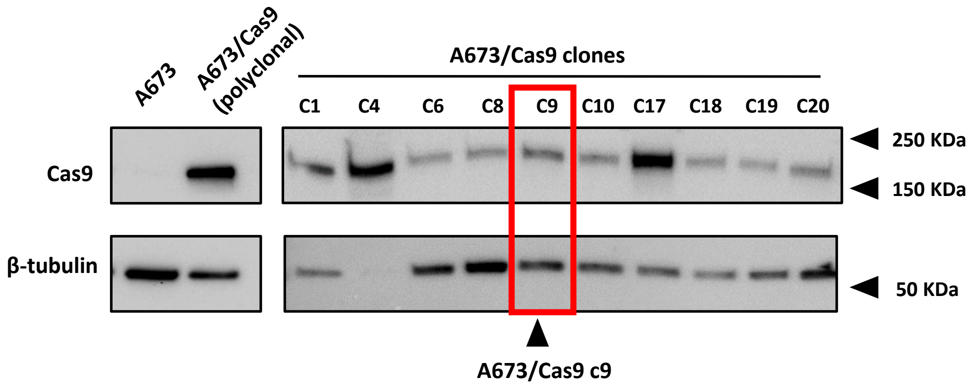

Figure S1. Establishment of A673/Cas9 cells. A673 cells were infected with CP-LvC9NU-02 vector and individual cell clones were isolated by limiting dilution procedure. Cas9 expression was analyzed by western-blot; polyclonal cell population (left) and monoclonal cell lines (right). The selected A673/Cas9 cell clone is indicated.

Figure S2

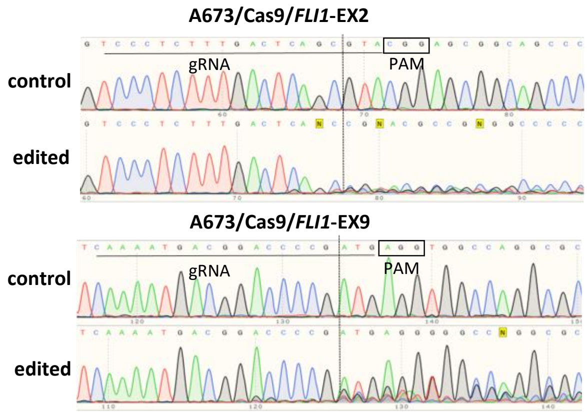

Figure S2. Confirmation of gene editing at the specified locus in A673/Cas9 cells infected with sgRNAs. Genomic DNA was PCR-amplified and each amplicon subjected to Sanger sequencing. Figure shows examples of Sanger electropherograms of A673/Cas9/FLI1-EX2 and FLI-EX9 cells. In both cases, overlapping Sanger traces starting near the Cas9 cut site were observed confirming the occurrence of gene editing. gRNA and PAM sequences are indicated

Supplementary figures

Figure S3

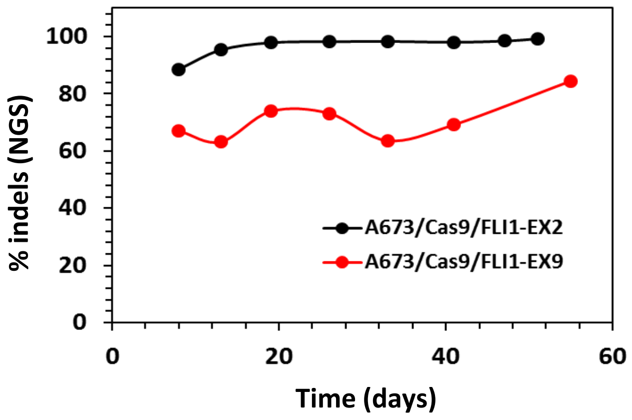

Figure S3. Quantification of gene editing rate at different time points. The figure shows the percentage of indels mutations quantified at each time point. Amplicons covering the target sequence were sequenced at a mean depth of around 20,000x and analyzed with an in-house software (Mosaic Finder) which allows us to identify the different DNA species present in the samples and study its evolution over time.

Figure S4 (a)

A673/Cas9/FLI1-EX2 (day 8)

```
WT                     GACGACCAGTCCCTCTTTGACTCAGC-GTACGGAGCGGCAGCCCATCTCCCCAAGGCCGACATGACTGCCTCGGGGA
insC  MUT1 (5.4%)      GACGACCAGTCCCTCTTTGACTCAGCCGTACGGAGCGGCAGCCCATCTCCCCAAGGCCGACATGACTGCCTCGGGGA
del9  MUT2 (2.5%)      GACGACCAGTCCCTCTTTGACTCAGC----------GGCAGCCCATCTCCCCAAGGCCGACATGACTGCCTCGGGGA
del2  MUT3 (2.4%)      GACGACCAGTCCCTCTTTGACTCA---GTACGGAGCGGCAGCCCATCTCCCCAAGGCCGACATGACTGCCTCGGGGA
del15 MUT4 (2.2%)      GACGACCAGTCCCTCTTTGACTCAGC----------------CCATCTCCCCAAGGCCGACATGACTGCCTCGGGGA
```

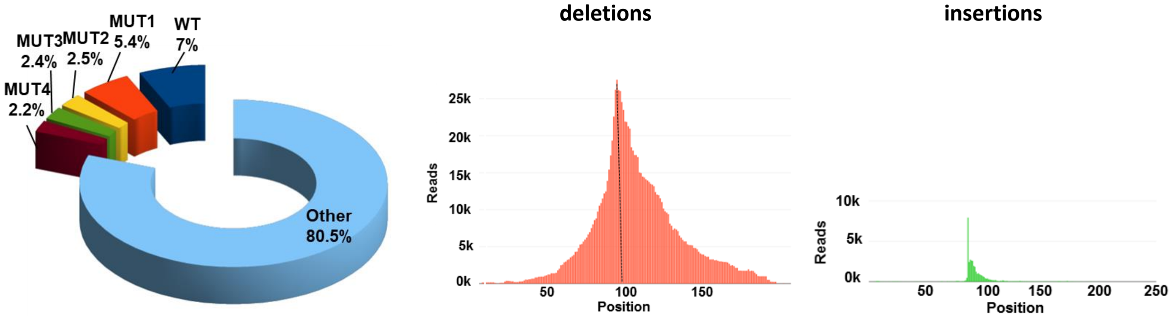

A673/Cas9/FLI1-EX2 (day 51)

```
WT                     GACGACCAGTCCCTCTTTGACTCAGC-GTACGGAGCGGCAGCCCATCTCCCCAAGGCCGACATGACTGCCTCGGGGA
insC  MUT1 (5.6%)      GACGACCAGTCCCTCTTTGACTCAGCCGTACGGAGCGGCAGCCCATCTCCCCAAGGCCGACATGACTGCCTCGGGGA
del9  MUT2 (3.0%)      GACGACCAGTCCCTCTTTGACTCAGC----------GGCAGCCCATCTCCCCAAGGCCGACATGACTGCCTCGGGGA
del2  MUT3 (2.8%)      GACGACCAGTCCCTCTTTGACTCA---GTACGGAGCGGCAGCCCATCTCCCCAAGGCCGACATGACTGCCTCGGGGA
del15 MUT4 (2.2%)      GACGACCAGTCCCTCTTTGACTCAGC----------------CCATCTCCCCAAGGCCGACATGACTGCCTCGGGGA
del7  MUT5 (1.4%)      GACGACCAGTCCCTCTTTGACTCAG--------AGCGGCAGCCCATCTCCCCAAGGCCGACATGACTGCCTCGGGGA
del44 MUT6 (1.3%)      GACGACCAGTCCCTCTTTGACT---------------------------------------------GCCTCGGGGA
del4  MUT7 (1.0%)      GACGACCAGTCCCTCTTTGACTCAGC-----GGAGCGGCAGCCCATCTCCCCAAGGCCGACATGACTGCCTCGGGGA
```

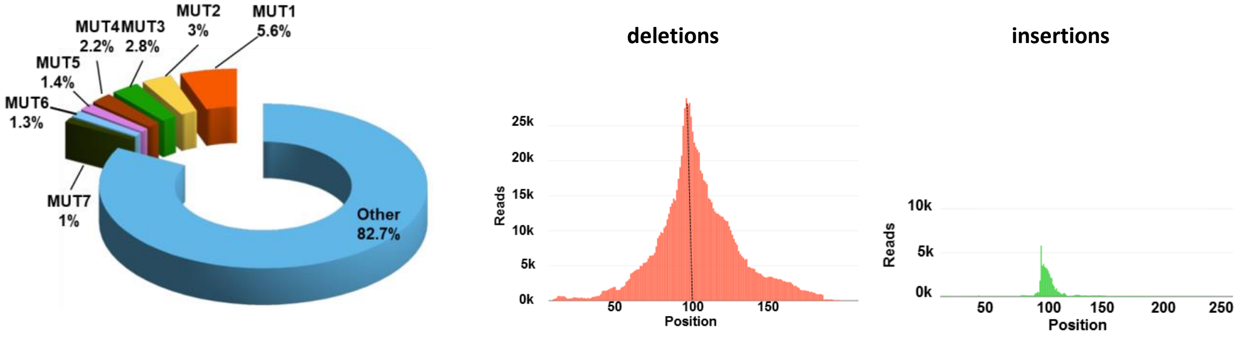

Figure S4. Detailed analysis of Cas9-mediated gene editing. DNA fragments covering the gRNA target were amplified by PCR and sequenced by NGS. Fastq files were analyzed with Mosaic Finder software in order to accurately determine mutation frequencies and gene editing efficiency at each nucleotide postition. DNA sequences of mutations present in a frequency greater than 1% are displayed (PAM sequence is highlighted). The distribution charts show the number of reads that harbor an insertion or deletion at each nucleotide position around the cut site predicted for Cas9 nuclease (vertical dotted line). All mutations above 1%, except one identified in A673/Cas9/FLI1-EX2 (a) and one identified in A673/Cas9/FLI1-EX9 (b) were predicted to produce frameshift and thus non-functional truncated proteins.

Cervera et al.

Figure S4 (b)

A673/Cas9/FLI1-EX9 (day 8)

```
WT                     ATCACCTGGGAGGGGACCAACGGGGAGTTCAAAATGACGGACCCCG-ATGAGGTGGCCAGGCGCTGGGGCGAGCGGA
del13 MUT1  (4.6%)     ATCACCTGGGAGGGGACCAACGGGGAGTTCAAAATGA--------------GGTGGCCAGGCGCTGGGGCGAGCGGA
del35 MUT2  (3.7%)     ATCACCTGGGAGGGGACCAACGGGGAGTTCAAAATGA------------------------------------CGGA
del?  MUT3  (2.1%)     ATCACCTGGG-------------------------------------------------------------------
del52 MUT4  (1.6%)     ATCACCTGGGAGGGGACGAGCG-----------------------------------------------------GA
del35 MUT5  (1.6%)     ATCACCTGGGAGGGGACCAACGGGGAGTTCAAAATGGCG------------------------------------GA
del28 MUT6  (1.3%)     ATCACCTGGGAGGGGACCAACGGGGAGTTCAAAATGACGGACCCC------------------------------GGA
del43 MUT7  (1.0%)     ATCACCTGGGAGGGGACCAACGGGGAGTTC--------------------------------------------GGA
```

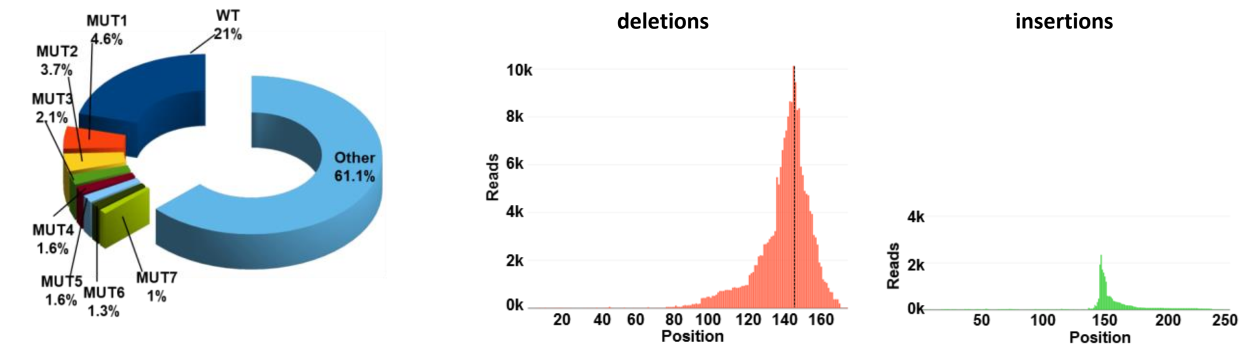

A673/Cas9/FLI1-EX9 (day 55)

```
WT                     ATCACCTGGGAGGGGACCAACGGGGAGTTCAAAATGACGGACCCCG-ATGAGGTGGCCAGGCGCTGGGGCGAGCGGA
del13 MUT1  (6.1%)     ATCACCTGGGAGGGGACCAACGGGGAGTTCAAAATGA--------------GGTGGCCAGGCGCTGGGGCGAGCGGA
del12 MUT8  (1.5%)     ATCACCTGGGAGGGGACCAACGGGGAGTTCAAAATGACGGACCCC--------------AGGCGCTGGGGCGAGCGGA
del14 MUT9  (1.4%)     ATCACCTGGGAGGGGACCAACGGGGAGTTCAAAATGACGGA---------------CCAGGCGCTGGGGCGAGCGGA
del52 MUT10 (1.3%)     ATCACCTGGGAGGGGAC-----------------------------------------------------GAGCGGA
insT  MUT11 (1.1%)     ATCACCTGGGAGGGGACCAACGGGGAGTTCAAAATGACGGACCCCGTATGAGGTGGCCAGGCGCTGGGGCGAGCGGA
insC  MUT12 (1.1%)     ATCACCTGGGAGGGGACCAACGGGGAGTTCAAAATGACGGACCCCGCATGAGGTGGCCAGGCGCTGGGGCGAGCGGA
del31 MUT13 (1.1%)     ATCACCTGGGAGGGGACCAACGGGGAGTTCAAAATG--------------------------------GCGAGCGGA
del2+31     14 (1.0%)  ATCACCTGGGAGGGGACCAA--GGGAGTTCAAAATGAC--------------------------------GAGCGGA
insGdel3    15 (1.0%)  ATCACCTGGGAGGGGACCAACGGGGAGTTCAAAATGACGGACCCCGG---AGGTGGCCAGGCGCTGGGGCGAGCGGA
del9+4      16 (1.0%)  ATCACCTGGGAGGGGACCAACGGGGAGTTCAAAATGACGGA----------GGTGGCCAGGCGCTGGGGC-----GA
```

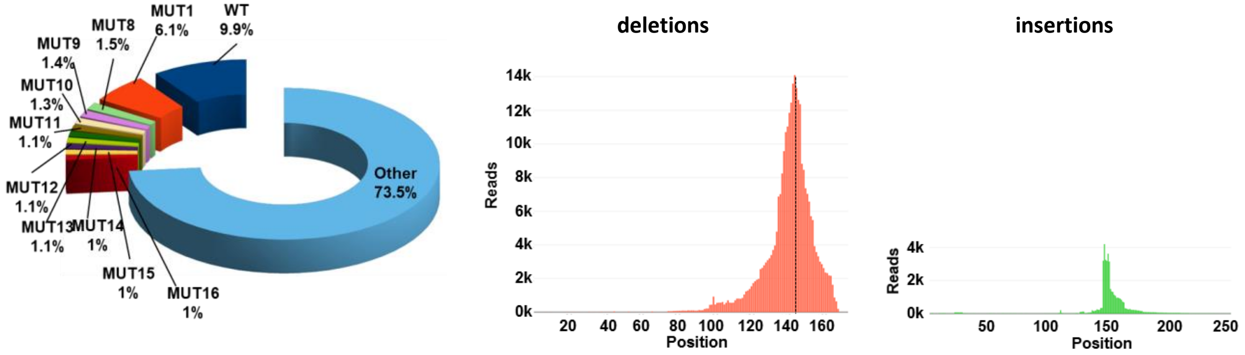

Supplementary figures

Figure S5

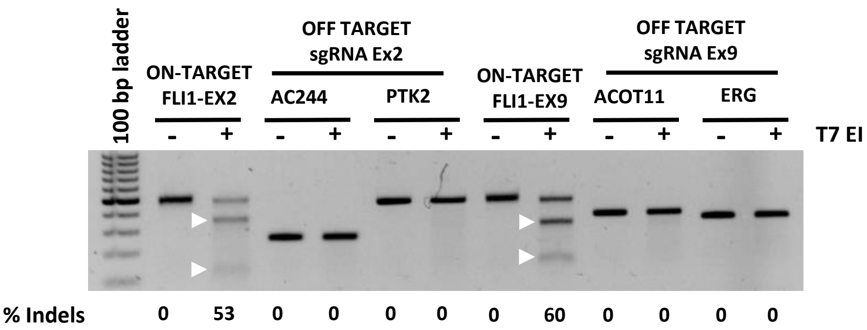

Figure S5. Off-target analysis. BreakingCas tool was used to identify putative off targets for gRNA sequences. For each gRNA, we selected two putative off-targets that ranked at the top of the list: AC244230.2 and PTK2 for gRNA Exon 2 and ACOT11 and ERG for gRNA Exon 9. Although these putative off-targets were the ones that got a higher ranking, their scores were already low (scores=0.4-0.8) compared to on target gRNAs (score=100). PCR products were amplified from genomic DNA with specific primers to the targeted region and then subjected to T7 endonuclease I (T7 EI) assay to detect gene editing. Gene editing was confirmed in on-target regions (i.e. amplicons were digested by T7EI generating two fragments; arrowheads) but not in off-target regions.
